# Mitochondrial bioenergetic changes during development as an indicator of *C. elegans* health-span

**DOI:** 10.18632/aging.102208

**Published:** 2019-08-27

**Authors:** Silvia Maglioni, Danielle F. Mello, Alfonso Schiavi, Joel N. Meyer, Natascia Ventura

**Affiliations:** 1 IUF-Leibniz Research Institute for Environmental Medicine, 40225 Duesseldorf, Germany; 2 Nicholas School of the Environment and Integrated Toxicology and Environmental Health Program, Duke University, Durham, NC 27708, USA; 3 Institute for Clinical Chemistry and Laboratory Diagnostic, Medical Faculty of the Heinrich Heine University, 40225 Duesseldorf, Germany

**Keywords:** mitochondria, mitochondrial respiration, Bioenergetic Health Index, *Caenorhabditis elegans*, lifespan, seahorse XF24 ^e^

## Abstract

Mild suppression of mitochondrial activity has beneficial effects across species. The nematode *Caenorhabditis elegans* is a versatile, genetically tractable model organism widely employed for aging studies, which has led to the identification of many of the known evolutionarily conserved mechanisms regulating lifespan. In *C. elegans* the pro-longevity effect of reducing mitochondrial function, for example by RNA interference, is only achieved if mitochondrial stress is applied during larval development. Surprisingly, a careful analysis of changes in mitochondrial functions resulting from such treatments during the developmental windows in which pro-longevity signals are programmed has never been carried out. Thus, although the powerful *C. elegans* genetics have led to the identification of different molecular mechanisms causally involved in mitochondrial stress control of longevity, specific functional mitochondrial biomarkers indicative or predictive of lifespan remain to be identified. To fill this gap, we systematically characterized multiple mitochondrial functional parameters at an early developmental stage in animals that are long-lived due to mild knockdown of twelve different mitochondrial proteins and correlated these parameters with animals’ lifespan. We found that basal oxygen consumption rate and ATP-linked respiration positively correlate with lifespan extension and propose the testable hypothesis that the Bioenergetic Health Index can be used as a proxy to predict health-span outcomes.

## Introduction

Mitochondria are of vital importance for survival: they are the cellular powerhouses, providing the necessary fuel for all biological processes, and carry out a wide range of other functions. It is therefore not surprising that severe deficiency of different mitochondrial proteins is associated with devastating consequences across species. Interestingly, it has been repeatedly shown that mild decrease in the expression or activity of the same mitochondrial proteins can lead instead to beneficial effects, such as increased resistance to stress, health improvement, and lifespan extension, in an evolutionarily conserved manner [1]. These provocative findings imply the existence of compensatory mitochondrial stress responses, or mitochondrial stress-triggered signaling events, which could potentially be exploited for targeted disease treatments and anti-aging strategies. The nematode *C. elegans* is a pioneer model organism in the aging field, and all molecular mechanisms causally involved in mitochondrial stress control of longevity have been identified thanks to its powerful genetics. These include a handful of transcription factors, autophagy- and apoptosis-regulatory genes, some kinases, as well as some mitochondrial metabolites and chromatin remodeling genes [2,3]. This class of long-lived, stress resistant *C. elegans* mutants, the so-called mitochondrial mutants (*Mit* mutants), is defined by genetic or RNAi-mediated suppression of genes directly or indirectly implicated in the functionality of the Mitochondrial Respiratory Chain (MRC) [4,5]. Different phenotypes that frequently accompany the lifespan extension of these *Mit* mutants include: changes in animal metabolism; the induction of protective and detoxifying systems (*e.g.,* mtUPR, antioxidant, autophagy and apoptosis pathways); smaller germline associated with decreased but prolonged fertility; and reduced adult size [3,6,7]. Of note, lifespan extension in the *Mit* mutants is only achieved if mitochondrial stress is applied during larval development [6,8], but the mechanism underlying this effect is unknown. Surprisingly, a careful analysis of mitochondrial function in the *Mit* mutants has not been carried out. Although a handful of publications investigated specific mitochondrial-related parameters (i.e., shape, volume, distribution, mitochondrial membrane potential, ROS production, ^mt^UPR induction) in young adult *C. elegans*, no meaningful correlations with lifespan were found [9–12]. However, direct analysis of the primary function of mitochondria - oxygen consumption related to oxidative phosphorylation - has not been carried out. Most notably, because previous analyses have assessed these mitochondrial parameters only in the adult stage, changes occurring during early developmental larval stages, which could be relevant for lifespan extension, would have been missed. Therefore, in this report, we systematically characterized mitochondrial functional parameters in different long-lived RNAi-mediated *Mit* mutants during an early developmental stage in which the lifespan benefits are most likely programmed, and correlated them with animals’ lifespan. Interestingly, we found that basal Oxygen Consumption Rate (OCR) and ATP-linked OCR positively correlate with lifespan extension. Furthermore, we propose that the derived Bioenergetic Health Index can be used as a proxy to predict health-span outcomes (Figure 4, graphic abstract).

## RESULTS

**Figure 1:**
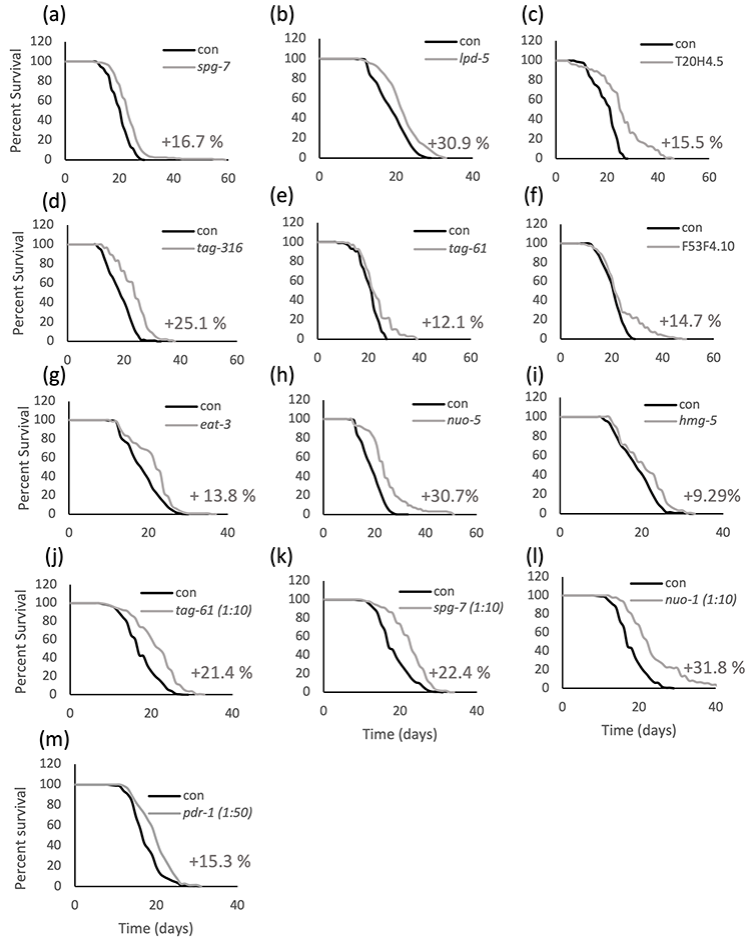
**Lifespan is extended upon mild suppression of different mitochondrial proteins.** Kaplan-Meier survival curves of wild-type animals fed bacteria transformed with empty vector pL4440 (con) or with pL4440 vector expressing dsRNA targeting the indicated mitochondrial proteins. Mild suppression of the mitochondrial proteins is achieved in the parental generation of animals using undiluted (a-i) or diluted (j-m) RNAi. % mean lifespan increase compared to control is shown next to the corresponding curve. An average of 60 animals per condition was used in each replica and survival curves of pooled population of animals coming from two to four independent replicas are shown. Refer to Supplementary Table SI for a complete statistical analysis of the lifespan assays.

Mild mitochondrial stress through genetic or pharmacological interventions extends *C. elegans* lifespan, while severe mitochondrial dysfunction, as expected from a disease state, has deleterious consequences ranging from arrested development to shortening of lifespan [2,3,6,12]. A screen performed in our laboratory in the past years to develop new *C. elegans* models for human mitochondrial-associated disorders (Maglioni et al., in preparation) further supported these earlier findings and led to the identification of different mitochondrial proteins whose severe RNAi-induced suppression caused detrimental phenotypes (i.e., arrested development, sterility or lethality) while extending lifespan upon mild RNAi suppression. Briefly, we screened 42 dsRNA clones selected to silence homologs of genes involved in human diseases for two generations, in search of genes whose suppression gave the characteristic phenotypic effects associated with different degrees of mitochondrial stress: a “mild” gene suppression causing slight decrease of size and slow development, and a “strong” gene suppression inducing sterility, developmental arrest, or lethality (Figure S1). Lifespan was subsequently measured for 25 clones that caused a mild decrease in growth, and for 11 of these we observed a significant extension of lifespan, ranging from 5 to 30% (Figure 1; Supplementary Table SI for statistical analysis). The remaining 14 genes gave either inconsistent results in the survival assay (3 out of 14 clones) or insignificant or negative lifespan extension, compared to control animals. Of note, most of the genes that did not extend lifespan also didn’t produce clearly distinct mild and strong phenotypes (Supplementary Table SII). For the purpose of this study, we decided to follow up on the 8 clones satisfying two criteria: 1) giving consistent results on survival analysis, in 3 independent replicas (or a high % increase even if only two replicas where carried out, *tag-316*), and 2) exhibiting clear reproducible phenotypic effects (mild vs strong). To increase the sample size, we included additional mitochondrial dsRNA clones from previous studies adhering to these criteria (i.e., *nuo-2*, *isp-1*, *atp-3, cco-1*) [13,14].

In the attempt to identify early larval changes in a mitochondrial respiration-associated parameter that specifically correlate with lifespan extension, we used the Seahorse Bioscience Flux Analyzer [15], after optimizing the protocols for nematodes at the L3 larval stage. To this end, synchronized wild-type (N2) worms were grown at 20°C on bacteria expressing dsRNA against different genes of interest (*tag-316,* F53F4.10, T20H4.5*, nuo-1, tag-61, atp-3, isp-1, cco-1, nuo-2, nuo-5*, *lpd-5* and *spg-7*), and mitochondrial respiration from these animals was compared with stage-matched, control animals (fed empty-vector transformed bacteria). We observed differentially altered mitochondrial profiles among the different RNAi treated nematodes, and could not identify a unifying trend in the alteration of the different respiratory parameters (i.e., no parameter was systematically only increased or decreased in all cases; Figure S2). Moreover, mild mitochondrial stress did not significantly affect either mitochondrial DNA abundance (Figure S3a) or mitochondrial DNA damage in a subset of the clones under study (Figure S3b).

**Figure 2:**
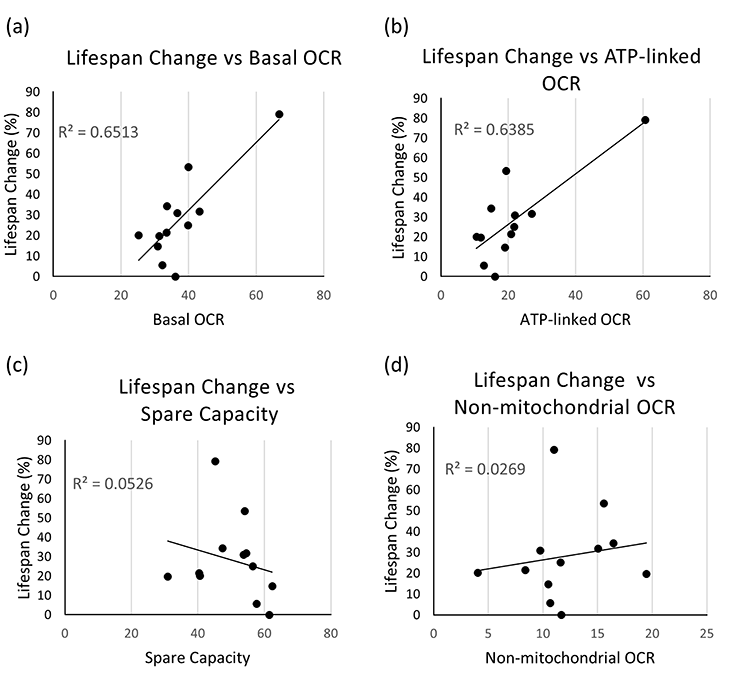
**Correlation between mitochondrial respiration and healthspan parameters.** (**a-d**) Correlation plots are shown between % increase in mean lifespan and basal OCR (**a**), ATP-linked OCR (**b**), spare capacity or SRC (**c**), and non-mitochondrial OCR (**d**). Each dot indicates an average value obtained from independent experiments with variable populations for each condition. For lifespan experiments, an average of 60 animals per condition per replica was used (in two to four independent replicates), while for Seahorse experiments an average of 1500 animals per condition per replica was assessed (at least 4 independent replicates per clone were carried out). Refer to Supplementary Table SI for data used to generate these panels.

Nonetheless, interestingly, when we looked at the possibility that mitochondrial bioenergetics-associated parameters during development correlate with lifespan extension, thus possibly being exploitable as predictor of life expectancy, we found a very strong positive relationship between lifespan changes (% increase compared to control animals) and basal OCR (Pearson correlation coefficient R=0.81, with R^2^=0.65 and a p-value=0.00078) (Figure 2a). Plotting the lifespan change against ATP-linked OCR similarly gave very strong positive correlation (R=0.80 with R^2^=0.63 and a p-value=0.00102) (Figure 2b). All other comparisons showed a negligible relationship (R^2^ ≤0.05 for lifespan vs spare respiratory capacity or SRC, non-mitochondrial OCR, and proton leak, and p-values = 0.47, 0.61, and 0.97, respectively) (Figures 2c-d and S3c). When we carried out statistical correlation analysis calculating Spearman’s correlation instead of Pearson correlation coefficients, the results were very similar (lifespan change against basal OCR R=0.71, with p-value=0.0065, lifespan change against ATP-linked OCR R=0.60 with a p-value=0.0301), demonstrating that the outliers are not substantially affecting the results.

**Figure 3:**
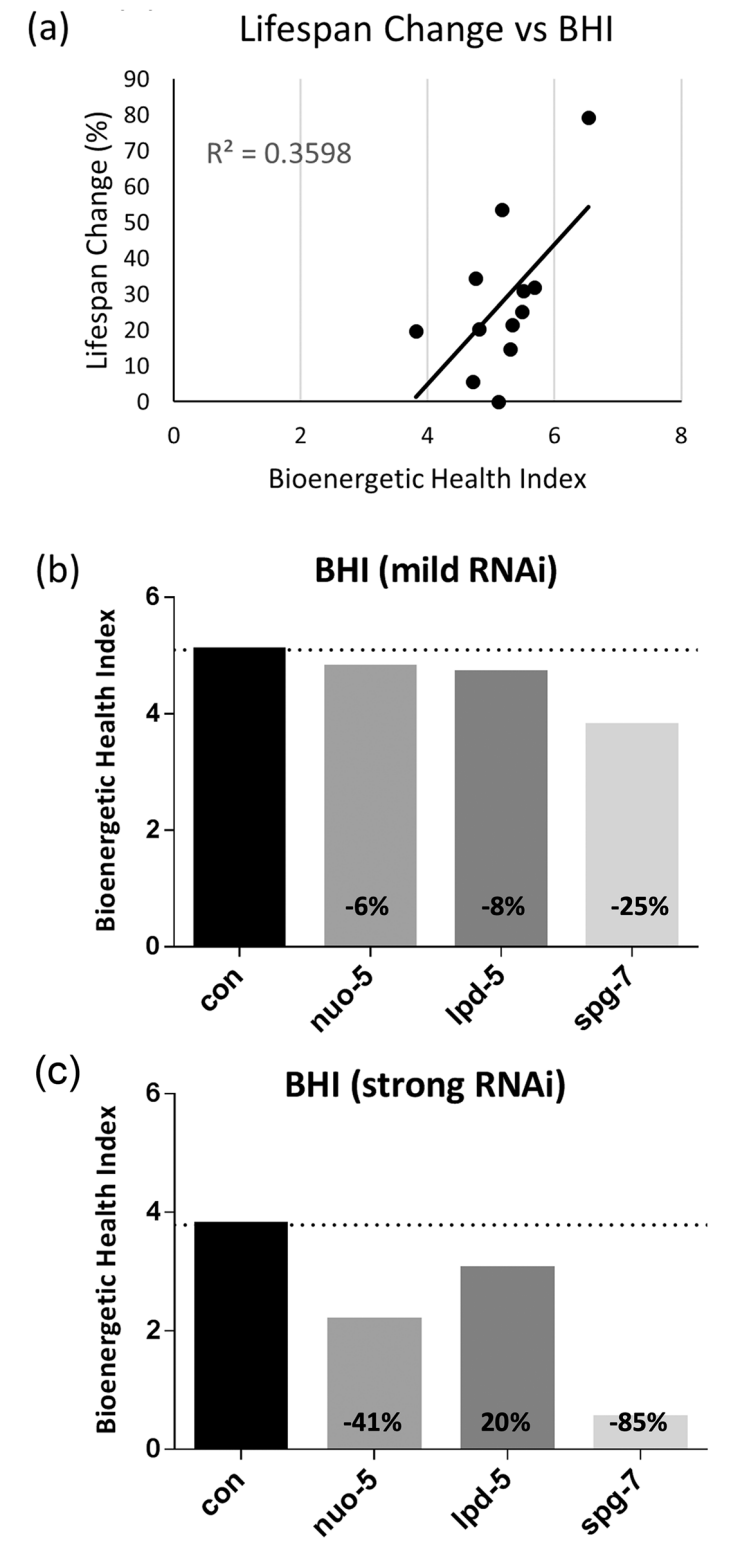
**Bioenergetic Health Index (BHI) calculation and correlation with lifespan extension.** BHI either (**a**) plotted against % increase in mean lifespan for all tested RNAi; or (**b**) calculated for mild (parental generation) and (**c**) strong (first generation) RNAi-mediated suppression of *nuo-5, lpd-5* and *spg-7.* % decrease of BHI compared to control is shown on the corresponding bar.

The Bioenergetic Health Index (BHI) is a single value that can be calculated from the different mitochondrial parameters obtained from the Seahorse XF Extracellular Flux Analyzer, using the equation proposed in Chacko et al. [16]. This parameter has been proposed to represent a composite mitochondrial profile indicating the health status for a selected cell type [16], and was suggested as a functional biomarker for oxidative stress in patients with metabolic disorders [17]. We used the respiration parameters shown in Figure S2 to calculate the BHIs for all the mild RNAi treatments (Figure S3d). When we plotted these values against the lifespan changes, we found a strong positive correlation (Pearson correlation coefficient of R=0.59, with R^2^=0.35 and a p-value=0.0338) (Figure 3a). We next wondered whether the BHI could provide any meaningful information regarding the health status of the worms in response to different degrees of mitochondrial stress. To test this hypothesis, we selected three genes (*spg-7*, *nuo-5* and *lpd-5*) whose mild silencing extends lifespan, while severe silencing leads to long-lived but developmentally-arrested animals (Figure S1 and S4). We found that while mild suppression of *spg-7*, *nuo-5* and *lpd-5* (and of the other clones under study) never reduced the BHI by more than 25% (Figure 3b and S3d), their strong suppression decreased it by up to 85% compared to wild type (Figure 3c). Of note, this also correlates with the severity of the RNAi-induced phenotype, with mild *spg-7* RNAi leading to small and pale but fertile animals, which live longer and have a BHI reduction of 25% compared to wild-type. On the other hand, the strong treatment induced developmental arrest as extremely small and sick L2/L3 larvae, and a BHI decrease of 85% compared to controls. Thus, at least in the case of these mitochondrial proteins, the BHI as weighted in this work represents an excellent predictor of animal health in response to mitochondrial stress. Although SRC did not strongly correlate with lifespan extension, we assigned the most weight to SRC and ATP-linked OCR (2 and 3 respectively), because we think these are the most relevant parameters that matter for health and lifespan. Indeed, we reason that SRC reflects the ability to respond to stress with increased energy production, and ATP-linked OCR reflects the baseline consumption of oxygen, analogous to a “rate of living” perspective of aging. We initially assigned the lowest value to non-mitochondrial OCR and proton leak because their relevance to lifespan is unclear, and these two parameters showed the greatest variance among the different replicates. However, one could argue that proton leak is, in fact, relevant to lifespan, given its potential role in altering ROS production. Contradictory results have been obtained for the relationship between proton leak and ROS generation [40–44], complicating predictions about its possible relevance in life- and health-span determination. Thus, in order to empirically test whether giving proton leak more weight would alter our results, we also determined the BHI giving a weight of 2 to this parameter. This change (Figure S5) did not substantially alter the correlation of BHI with lifespan, but further reduced the values obtained for BHI upon severe RNAi, thus supporting a role for proton leak in determining health-span rather than lifespan.

A closer analysis of mitochondrial respiration in the *spg-7* knockdown L3 larvae revealed that basal OCR, maximal OCR, ATP-linked OCR and SRC are all slightly reduced in the long-lived *spg-7* mild RNAi-treated animals, but are much more significantly reduced in the arrested, sick animals resulting from strong *spg-7* suppression (Figure S6a-d). Proton leak is not altered upon mild suppression, but greatly increased upon strong suppression (Figure S6e), and non-mitochondrial OCR is never altered (Figure S6f). Collectively, these results suggest that the functional impacts of the lifespan-extending mitochondrial inhibitions are quite mild, indicating a certain metabolic flexibility is required to promote pro-longevity cellular reprogramming, while stronger functional impacts, as expected, are deleterious.

## DISCUSSION

**Figure 4:**
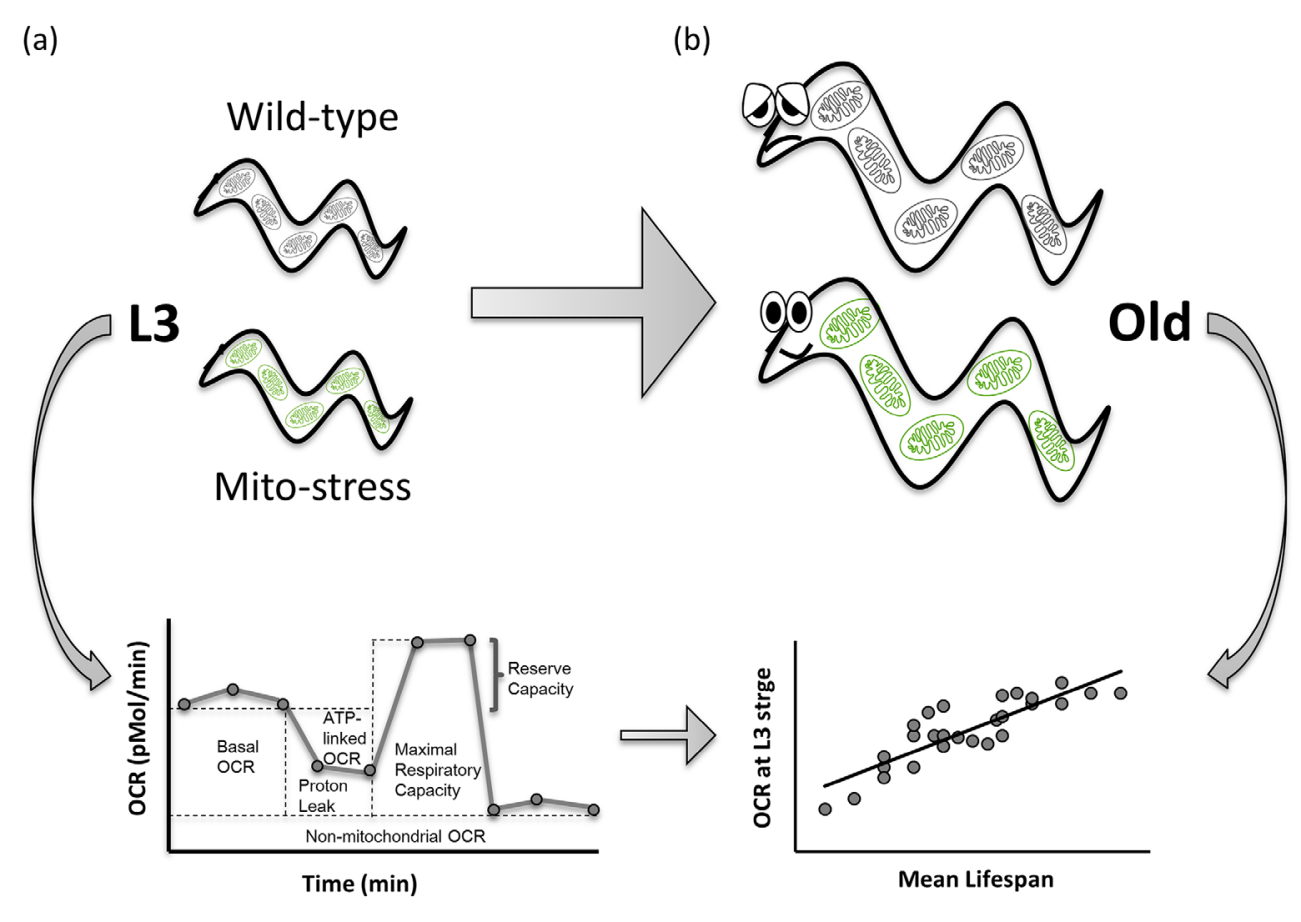
**Basal OCR and ATP-linked OCR measured during development positively correlate with lifespan extension in *C. elegans* mitochondrial mutants.** Larval development is a critical stage for pro-longevity reprogramming upon mitochondrial stress in *C. elegans.* Basal and ATP-linked Oxygen Consumption Rate, assessed at third larval stage (**a**) very strongly correlate with lifespan extension in long-lived RNAi-derived *Mit* mutants (**b**), representing a potential predictor of lifespan extension in response to mitochondrial stress.

In summary, we showed for the first time that alterations in basal and ATP-linked OCR (but not other specific mitochondrial parameters), in the critical third larval developmental stage, represent a potential predictor of lifespan extension in response to mitochondrial stress, and that these changes most likely precede pro-longevity reprogramming processes (Figure 4). Although changes in basal and ATP-linked OCR may simply correlate with lifespan extension, due to the observed very strong correlation, it is instead likely that these may promote metabolic, genetic and epigenetic reprogramming later in life [18–20], which are, in fact, causally involved in longevity specification. Thus, as opposed to the metabolic inflexibility associated with cellular senescence [21,22] and the detrimental effects observed across species at the cellular and organismal level upon severe mitochondrial dysfunction [23], mild mitochondrial stress leads to less profound alteration of mitochondrial functional parameters. In *C. elegans,* this appears to be associated with a certain degree of metabolic flexibility (possibly captured by the BHI), promoting anti-aging effects. Of note, evidence that partial reduction of mitochondrial function promotes healthy aging has also been collected in mammals. For instance, similar to the pro-longevity effect of *clk-1* mutants observed in *C. elegans,* hemizygous knockout of mouse clk1 (Mclk1) extended mice lifespan, and mclk1-/- embryonic stem cells displayed decreased ROS levels, ROS sensitivity, and ROS-induced damage [24]. Furthermore, genetic- or pharmacological-induced suppression of mitochondrial ribosomal subunits, with consequent mito-nuclear protein imbalance and activation of the mtUPR, was identified as a pro-longevity mechanism conserved in both *C. elegans* and mice [7]. Most notably, contrary to expectation, mice carrying a homozygous knockout of the cytochrome c assembly factor (Surf1) - a gene in which mutations cause Leigh syndrome - displayed significantly prolonged lifespan and enhanced memory, and were protected against Ca^2+^-dependent neurodegeneration [25,26]. It is therefore plausible that correlation studies may help identify mitochondrial parameters that would serve as early predictors of health-span in mammals. While no correlation between human vitality and mitochondrial respiratory parameters in peripheral blood mononuclear cells could be identified [27], looking at a systemic level or at cells/tissues strongly relying on mitochondrial metabolism may produce more compelling results.

The combination of multiple mitochondrial bioenergetics parameters into the BHI may especially serve this purpose, since it could, in fact, capture the metabolic flexibility of a cell under energy demand. Although this novel tool is not yet well validated, the BHI has been suggested as an indicator of cellular health in patients with metabolic disorders [16,17]. It has also been proposed as potential biomarker for changes in brain mitochondrial metabolism to be exploited as a screening tool to assess the risk of developing neurodegenerative diseases [28]. The BHI is calculated by assigning a specific weight to the different bioenergetics parameters based on their relative biological significance or pathological relevance (e.g., different weight may be assigned in different cell types) [16,17]. Here we adapted it to our system by weighting the different mitochondrial parameters based on the data we collected with the Seahorse and our assessment of their potential significance in the adaptive mitochondrial stress responses, and we proposed the BHI as a possible strong and reliable early indicator of animal health-span. Recently the BHI of single worms during aging was also calculated, and it was shown to reach its maximum in 4-day old worms and then decline [29]. The authors proposed to use the BHI as a dynamic index to show the status of the bioenergetic health of *C. elegans* during growth and aging processes. It will be interesting to verify whether alteration of the BHI can indeed be exploited as a proxy indicator of individual health and lifespan in response to different genetic or extrinsic perturbations.

## MATERIALS AND METHODS

### *C. elegans* strains and maintenance

We employed standard nematode culture conditions [30]. All nematodes were maintained at 20°C on Nematode Growth Media agar supplemented with Escherichia coli (OP50 or transformed HT115). Only the wild-type strain N2 was employed in this work; it was provided by the *Caenorhabditis* Genetics Center (CGC).

### RNAi feeding

The following dsRNA transformed bacteria for feeding were derived from the Ahringer *C. elegans* RNAi library [31] and sequence verified: *nuo-1* (C09H10.3), T20H4.5, F53F4.10, *cco-1* (F26E4.9), *nuo-5* (Y45G12B.1), *nuo-2* (T10E9.7), *spg-7* (Y47G6A.10), *lpd-5* (ZK973.10*) tag-316* (T01B11.4), *tag-61* (T27E9.1), *atp-3* (F27C1.7)*, sco-1* (C01F1.2), *sod-1* (C15F1.7), *cox-10* (Y46G5A.2), *fum-1* (H14A12.2), *aco-2,* (F54H12.1)*, ymel-1* (M03C11.5), *drp-1* (T12E12.4), *ogdh-1* (T22B11.5) *, hmgs-1* (F25B4.6)*, fis-2* (F13B9.8), *sdha-1*(C03G5.1), *aldo-2* (F01F1.12), *dlst-1*(W02F12.5*), dlat-1* (F23B12.5). Feeding RNAi constructs against *isp-1* were generated as previously described [6,32]. Density of transformed HT115 bacteria was normalized to an OD_600_=0.9 before being used to seed RNAi plates (NGM/IPTG/Ampicillin/ Tetracycline) for all clones; however, *nuo-1*, *cco-1*, *nuo-5*, *nuo-2*, *spg-7*, *lpd-5*, *tag-61*, *atp-3*, *cco-1* and *isp-1* clones were diluted 1:10 (or 1:50 when indicated) with empty vector pL4440-expressing bacteria (also normalized to OD_600_=0.9) as previously described [6]. Briefly, most of the clones screened showed a "mild" phenotype when the parental generation was grown on undiluted bacteria (OD_600_=0.9), and a "strong" phenotype in the second generation. Some clones instead showed a "strong" phenotype in the parental generation, and in these cases the animals were fed with a diluted dsRNA-expressing bacterial clone to obtain a "mild" phenotype.

### Lifespan assay

Lifespan and statistical survival analysis were performed as previously described [13] using synchronous populations of at least 60 animals per strain. Survival analysis started from hatching and was carried out at 20°C. Animals were scored as dead or alive and transferred every day onto fresh plates during the fertile period, and then every other day or every 3 days until death. Worms were considered dead when they stop pharyngeal pumping and responding to touch. Worms that died because of internal bagging, desiccation due to crawling on the edge of the plates, or gonad extrusion were scored as censored. These animals were included in lifespan analyses up to the point of censorship and were weighted by half in the statistical analysis. We calculated mean lifespan, standard deviation of the mean, and P value (Mantel-Cox regression analysis) from Kaplan-Meyer survival curves of pooled population of animals coming from at least two independent replicates. For statistical analysis we used the Online Application for Survival analysis OASIS 2 [33]. See Table S1 for a summary of lifespan data.

### Measurement of *C. elegans* mitochondrial functional parameters

The fundamental parameters of mitochondrial respiratory chain, including basal oxygen consumption rate (OCR), maximal respiratory capacity, SRC, ATP coupled OCR, proton leak, and non-mitochondrial OCR were assessed with pharmacological inhibitors of the electron transport chain (ETC) using the Seahorse XFe24 Extracellular Flux Analyzer (XFe; Seahorse Bioscience, Massachusetts, USA) according to Luz et al. [15,34], except that we used the 24-well Seahorse XF24 V7 PS Cell Culture microplates, instead of islet plates. Briefly, synchronized wild-type (*N2)* embryos were obtained by sodium hydroxide bleach treatment of gravid adults [35], added to RNAi plates containing bacteria expressing dsRNA against the genes of interest, and grown at 20°C until the L3 larval stage (38-41h). We chose this stage, as the “strong” treatment with RNAi against some of the mitochondrial proteins under study (e.g., *nuo-5*, *lpd-5 and spg-7*) lead to arrest animal development at L2/L3 larval stage. Animals submitted to the “mild” RNAi treatment were grown in RNAi plates for one generation, whereas for the “strong” RNAi treatment, they were grown for two consecutive generations. Mitochondrial parameters from these animals were compared with stage-matched animals grown on bacteria expressing the empty vector pL4440. Worms were rinsed off the RNAi plates, washed, placed at 20 °C on a rocker for 20 minutes to allow them to clear their guts of bacteria, washed once more, and then resuspended in unbuffered EPA water [15]. Following, animals were counted and 250 nematodes were pipetted into each well. The final volume of each well was brought to 525 μl with unbuffered EPA water. Two wells per assay were filled with EPA water without worms and used as blanks. The inhibition of ATP synthase by Dicyclohexylcarbodiimide (DCCD) provided a measure of the amount of oxygen consumption coupled to ATP production (ATP-linked OCR). Uncoupling ATP production from oxygen consumption with carbonyl cyanide 4-(trifluoromethoxy) phenylhydrazone (FCCP) provided a measure of maximal respiratory capacity. By subtracting from this value the basal OCR we calculated the SRC, an indication of an organism’s ability to respond to increased energy demands. Finally, OCR after injecting the cytochrome c oxidase inhibitor sodium azide (NaAz) provided a measure of non-mitochondrial respiration, and by subtracting the azide response from the DCCD-inhibited OCR we calculated proton leak. 75 μL of each drug were loaded into the designated injection ports of the seahorse cartridge. The final solution concentrations after injection of each drug was the following: 20 μM DCCD (1% DMSO), 25 μM FCCP (2% DMSO), and 10 mM azide. Two runs were performed per day with worms harvested from RNAi plates at 38h for the first run and at 41h for the second run. In both runs wells from half of the plate received injections of DCCD followed by NaAz, and the other half received FCCP injections only. Basal OCR measurements were always performed before the injection of the mitochondrial inhibitors. OCR values were normalized to the actual number of worms in each well and the values obtained were again normalized to the protein content per worm measured with a BCA protein assay of a subset of the same worms used for OCR measurement. ~2000 nematodes were sonicated for each condition (for a detailed worm protein measurement protocol see Luz et al. 2015).

### Statistical analysis of mitochondrial functional parameters

Seahorse experiments were repeated at least four times. Between 4 and 6 wells per 24-well plate were used for each RNAi treatment in each replicate. Because of the variability of the OCR measurements among plates, each parameter for each RNAi treatment was normalized as fold change compared to the control (i.e., OCR for empty vector pL4440 was set to one) in each plate. The fold changes of all single experiments were then pooled together. For each respiration parameter, a mean value for the control (pL4440) was then calculated by averaging the actual values of controls from every plate. The fold changes of each clone were finally divided by these control means to obtain the Seahorse parameters reported in Table SI (in nmol O_2_/min/mg protein). Every mitochondrial parameter was initially assessed with a one-way ANOVA. A post-hoc analysis of between-group differences using the Student’s t-test was carried out when justified by significant effects observed in the one-way ANOVA.

### Analysis of mitochondrial DNA copy number and mitochondrial DNA damage

Synchronized wild-type (*N2)* worms were grown at 20°C on bacteria expressing dsRNA against the genes of interest*.* nDNA and mtDNA copy numbers from these animals were compared with stage-matched animals fed bacteria expressing the empty vector pL4440. Six worms were picked and pooled in a single tube per biological replicate, and three biological replicates were taken per treatment in three experiments separated in time. mtDNA damage was evaluated in *nuo-5, lpd-5* and *spg-7* knockdown worms using a quantitative PCR (qPCR)-based method as previously described [36]. This assay defines the control samples as undamaged and determines a lesion frequency in experimental samples based on any decrease in amplification efficiency relative to the control samples [37]. One nuclear genome target (9.3 kb) and one mitochondrial genome target (10.9 kb) were amplified. The DNA damage assay is able to quantitatively measure the number of polymerase-stalling lesions based on the amount of amplification obtained from the qPCR. To calculate DNA copy number we utilized a qPCR assay in which the sample cycle thresholds are compared to a standard [38,39]. This method is particularly advantageous because the actual number of genome copies can be calculated.

### Calculation of p values for the Pearson correlation coefficient r

A web-based calculator was used to obtain *p* values (http://www.socscistatistics.com/) for correlations in this study.

### Calculation of Bioenergetic Health Index

The Bioenergetic Health Index (BHI) was calculated based on the formula below:

BHI = logspare capacity^a x ATP-linked OCR^bnon-mitochondrial OCR^c x proton leak^d

Modifying the terms a, b, c, d in the equation one can attribute different weight to the different respiratory parameters. We used the values a=2, b=3, c=1, d=1 or 2 for our calculations.

## SUPPLEMENTARY MATERIAL

Supplementary Tables

Supplementary Figures

## References

[1] Hwang AB, Jeong DE, Lee SJ. Mitochondria and organismal longevity. Curr Genomics. 2012; 13:519–32. 10.2174/138920212803251427 23633912 PMC3468885

[2] Schiavi A, Ventura N. Mitochondrial Longevity Pathways, in Ageing: Lessons from C. elegans. 2017, Springer International Publishing Switzerland 2017.

[3] Munkácsy E, Rea SL. The paradox of mitochondrial dysfunction and extended longevity. Exp Gerontol. 2014; 56:221–33. 10.1016/j.exger.2014.03.016 24699406 PMC4104296

[4] Rea SL. Metabolism in the Caenorhabditis elegans Mit mutants. Exp Gerontol. 2005; 40:841–49. 10.1016/j.exger.2005.06.015 16137850

[5] Ventura N, Rea SL, Testi R. Long-lived C. elegans mitochondrial mutants as a model for human mitochondrial-associated diseases. Exp Gerontol. 2006; 41:974–91. 10.1016/j.exger.2006.06.060 16945497

[6] Rea SL, Ventura N, Johnson TE. Relationship between mitochondrial electron transport chain dysfunction, development, and life extension in Caenorhabditis elegans. PLoS Biol. 2007; 5:e259. 10.1371/journal.pbio.0050259 17914900 PMC1994989

[7] Houtkooper RH, Mouchiroud L, Ryu D, Moullan N, Katsyuba E, Knott G, Williams RW, Auwerx J. Mitonuclear protein imbalance as a conserved longevity mechanism. Nature. 2013; 497:451–57. 10.1038/nature12188 23698443 PMC3663447

[8] Dillin A, Hsu AL, Arantes-Oliveira N, Lehrer-Graiwer J, Hsin H, Fraser AG, Kamath RS, Ahringer J, Kenyon C. Rates of behavior and aging specified by mitochondrial function during development. Science. 2002; 298:2398–401. 10.1126/science.1077780 12471266

[9] Regmi SG, Rolland SG, Conradt B. Age-dependent changes in mitochondrial morphology and volume are not predictors of lifespan. Aging (Albany NY). 2014; 6:118–30. 10.18632/aging.100639 24642473 PMC3969280

[10] Bennett CF, Vander Wende H, Simko M, Klum S, Barfield S, Choi H, Pineda VV, Kaeberlein M. Activation of the mitochondrial unfolded protein response does not predict longevity in Caenorhabditis elegans. Nat Commun. 2014; 5:3483. 10.1038/ncomms4483 24662282 PMC3984390

[11] Dingley S, Polyak E, Lightfoot R, Ostrovsky J, Rao M, Greco T, Ischiropoulos H, Falk MJ. Mitochondrial respiratory chain dysfunction variably increases oxidant stress in Caenorhabditis elegans. Mitochondrion. 2010; 10:125–36. 10.1016/j.mito.2009.11.003 19900588 PMC3638869

[12] Ventura N, Rea SL. Caenorhabditis elegans mitochondrial mutants as an investigative tool to study human neurodegenerative diseases associated with mitochondrial dysfunction. Biotechnol J. 2007; 2:584–95. 10.1002/biot.200600248 17443764

[13] Ventura N, Rea SL, Schiavi A, Torgovnick A, Testi R, Johnson TE. p53/CEP-1 increases or decreases lifespan, depending on level of mitochondrial bioenergetic stress. Aging Cell. 2009; 8:380–93. 10.1111/j.1474-9726.2009.00482.x 19416129 PMC2730656

[14] Maglioni S, Schiavi A, Runci A, Shaik A, Ventura N. Mitochondrial stress extends lifespan in C. elegans through neuronal hormesis. Exp Gerontol. 2014; 56:89–98. 10.1016/j.exger.2014.03.026 24709340

[15] Luz AL, Smith LL, Rooney JP, Meyer JN. Seahorse Xfe 24 Extracellular Flux Analyzer-Based Analysis of Cellular Respiration in Caenorhabditis elegans. Curr Protoc Toxicol. 2015; 66:1–15. 10.1002/0471140856.tx2507s66 26523474 PMC4632645

[16] Chacko BK, Kramer PA, Ravi S, Benavides GA, Mitchell T, Dranka BP, Ferrick D, Singal AK, Ballinger SW, Bailey SM, Hardy RW, Zhang J, Zhi D, Darley-Usmar VM. The Bioenergetic Health Index: a new concept in mitochondrial translational research. Clin Sci (Lond). 2014; 127:367–73. 10.1042/CS20140101 24895057 PMC4202728

[17] Chacko BK, Zhi D, Darley-Usmar VM, Mitchell T. The Bioenergetic Health Index is a sensitive measure of oxidative stress in human monocytes. Redox Biol. 2016; 8:43–50. 10.1016/j.redox.2015.12.008 26748041 PMC4712317

[18] Mishur RJ, Khan M, Munkácsy E, Sharma L, Bokov A, Beam H, Radetskaya O, Borror M, Lane R, Bai Y, Rea SL. Mitochondrial metabolites extend lifespan. Aging Cell. 2016; 15:336–48. 10.1111/acel.12439 26729005 PMC4783347

[19] Tatar M, Sedivy JM. Mitochondria: masters of Epigenetics. Cell. 2016; 165:1052–54. 10.1016/j.cell.2016.05.021 27203109 PMC5383427

[20] Schiavi A, Maglioni S, Palikaras K, Shaik A, Strappazzon F, Brinkmann V, Torgovnick A, Castelein N, De Henau S, Braeckman BP, Cecconi F, Tavernarakis N, Ventura N. Iron-Starvation-Induced Mitophagy Mediates Lifespan Extension upon Mitochondrial Stress in C. elegans. Curr Biol. 2015; 25:1810–22. 10.1016/j.cub.2015.05.059 26144971

[21] Capasso S, Alessio N, Squillaro T, Di Bernardo G, Melone MA, Cipollaro M, Peluso G, Galderisi U. Changes in autophagy, proteasome activity and metabolism to determine a specific signature for acute and chronic senescent mesenchymal stromal cells. Oncotarget. 2015; 6:39457–68. 10.18632/oncotarget.6277 26540573 PMC4741838

[22] López-Otín C, Galluzzi L, Freije JM, Madeo F, Kroemer G. Metabolic Control of Longevity. Cell. 2016; 166:802–21. 10.1016/j.cell.2016.07.031 27518560

[23] Maglioni S, Ventura N. C. elegans as a model organism for human mitochondrial associated disorders. Mitochondrion. 2016; 30:117–25. 10.1016/j.mito.2016.02.003 26906059

[24] Liu X, Jiang N, Hughes B, Bigras E, Shoubridge E, Hekimi S. Evolutionary conservation of the clk-1-dependent mechanism of longevity: loss of mclk1 increases cellular fitness and lifespan in mice. Genes Dev. 2005; 19:2424–34. 10.1101/gad.1352905 16195414 PMC1257397

[25] Dell’agnello C, Leo S, Agostino A, Szabadkai G, Tiveron C, Zulian A, Prelle A, Roubertoux P, Rizzuto R, Zeviani M. Increased longevity and refractoriness to Ca(2+)-dependent neurodegeneration in Surf1 knockout mice. Hum Mol Genet. 2007; 16:431–44. 10.1093/hmg/ddl477 17210671

[26] Lin AL, Pulliam DA, Deepa SS, Halloran JJ, Hussong SA, Burbank RR, Bresnen A, Liu Y, Podlutskaya N, Soundararajan A, Muir E, Duong TQ, Bokov AF, et al. Decreased in vitro mitochondrial function is associated with enhanced brain metabolism, blood flow, and memory in Surf1-deficient mice. J Cereb Blood Flow Metab. 2013; 33:1605–11. 10.1038/jcbfm.2013.116 23838831 PMC3790931

[27] Maynard S, Keijzers G, Gram M, Desler C, Bendix L, Budtz-Jørgensen E, Molbo D, Croteau DL, Osler M, Stevnsner T, Rasmussen LJ, Dela F, Avlund K, Bohr VA. Relationships between human vitality and mitochondrial respiratory parameters, reactive oxygen species production and dNTP levels in peripheral blood mononuclear cells. Aging (Albany NY). 2013; 5:850–64. 10.18632/aging.100618 24304678 PMC3868727

[28] Tyrrell DJ, Bharadwaj MS, Jorgensen MJ, Register TC, Shively C, Andrews RN, Neth B, Keene CD, Mintz A, Craft S, Molina AJ. Blood-Based Bioenergetic Profiling Reflects Differences in Brain Bioenergetics and Metabolism. Oxid Med Cell Longev. 2017; 2017:7317251. 10.1155/2017/7317251 29098063 PMC5643153

[29] Huang SH, Lin YW. Bioenergetic Health Assessment of a Single Caenorhabditis elegans from Postembryonic Development to Aging Stages via Monitoring Changes in the Oxygen Consumption Rate within a Microfluidic Device. Sensors (Basel). 2018; 18:E2453. 10.3390/s18082453 30060586 PMC6111518

[30] Stiernagle T. Maintenance of C. elegans. WormBook. 2006; pp.1–11. 10.1895/wormbook.1.101.1 18050451 PMC4781397

[31] Kamath RS, Ahringer J. Genome-wide RNAi screening in Caenorhabditis elegans. Methods. 2003; 30:313–21. 10.1016/S1046-2023(03)00050-1 12828945

[32] Ventura N, Rea S, Henderson ST, Condo I, Johnson TE, Testi R. Reduced expression of frataxin extends the lifespan of Caenorhabditis elegans. Aging Cell. 2005; 4:109–12. 10.1111/j.1474-9726.2005.00149.x 15771615

[33] Han SK, Lee D, Lee H, Kim D, Son HG, Yang JS, Lee SV, Kim S. OASIS 2: online application for survival analysis 2 with features for the analysis of maximal lifespan and healthspan in aging research. Oncotarget. 2016; 7:56147–52. 10.18632/oncotarget.11269 27528229 PMC5302902

[34] Luz AL, Rooney JP, Kubik LL, Gonzalez CP, Song DH, Meyer JN. Mitochondrial Morphology and Fundamental Parameters of the Mitochondrial Respiratory Chain Are Altered in Caenorhabditis elegans Strains Deficient in Mitochondrial Dynamics and Homeostasis Processes. PLoS One. 2015; 10:e0130940. 10.1371/journal.pone.0130940 26106885 PMC4480853

[35] Boyd WA, Smith MV, Freedman JH. Caenorhabditis elegans as a model in developmental toxicology. Methods Mol Biol. 2012; 889:15–24. 10.1007/978-1-61779-867-2_3 22669657 PMC3513774

[36] Gonzalez-Hunt CP, Rooney JP, Ryde IT, Anbalagan C, Joglekar R, Meyer JN. PCR-Based Analysis of Mitochondrial DNA Copy Number, Mitochondrial DNA Damage, and Nuclear DNA Damage. Curr Protoc Toxicol. 2016; 67:1–25, 25. 26828332 10.1002/0471140856.tx2011s67 PMC4928199

[37] Meyer JN. QPCR: a tool for analysis of mitochondrial and nuclear DNA damage in ecotoxicology. Ecotoxicology. 2010; 19:804–11. 10.1007/s10646-009-0457-4 20049526 PMC2844971

[38] Rooney JP, Ryde IT, Sanders LH, Howlett EH, Colton MD, Germ KE, Mayer GD, Greenamyre JT, Meyer JN. PCR based determination of mitochondrial DNA copy number in multiple species. Methods Mol Biol. 2015; 1241:23–38. 10.1007/978-1-4939-1875-1_3 25308485 PMC4312664

[39] Venegas V, Halberg MC. Measurement of mitochondrial DNA copy number. Methods Mol Biol. 2012; 837:327–35. 10.1007/978-1-61779-504-6_22 22215558

[40] Brookes PS. Mitochondrial H(+) leak and ROS generation: an odd couple. Free Radic Biol Med. 2005; 38:12–23. 10.1016/j.freeradbiomed.2004.10.016 15589367

[41] Brand MD. Uncoupling to survive? The role of mitochondrial inefficiency in ageing. Exp Gerontol. 2000; 35:811–20. 10.1016/S0531-5565(00)00135-2 11053672

[42] Zhao RZ, Jiang S, Zhang L, Yu ZB. Mitochondrial electron transport chain, ROS generation and uncoupling (Review). Int J Mol Med. 2019; 44:3–15. Review 31115493 10.3892/ijmm.2019.4188 PMC6559295

[43] Sedensky MM, Morgan PG. Mitochondrial respiration and reactive oxygen species in mitochondrial aging mutants. Exp Gerontol. 2006; 41:237–45. 10.1016/j.exger.2006.01.004 16497463

[44] Cheng J, Nanayakkara G, Shao Y, Cueto R, Wang L, Yang WY, Tian Y, Wang H, Yang X. Mitochondrial Proton Leak Plays a Critical Role in Pathogenesis of Cardiovascular Diseases. Adv Exp Med Biol. 2017; 982:359–70. 10.1007/978-3-319-55330-6_20 28551798 PMC5630226

