# Supplementary material for: Mitochondrial bioenergetic changes during development as an indicator of C. elegans health-span
Source: Aging (Albany NY). 2019 Aug 27;11(16):6535–54. doi: 10.18632/aging.102208 (PMC6738431; doi:10.18632/aging.102208)
Supplement: Supplementary Figures [file aging-11-102208-s002.pdf]

SUPPLEMENTARY FIGURES

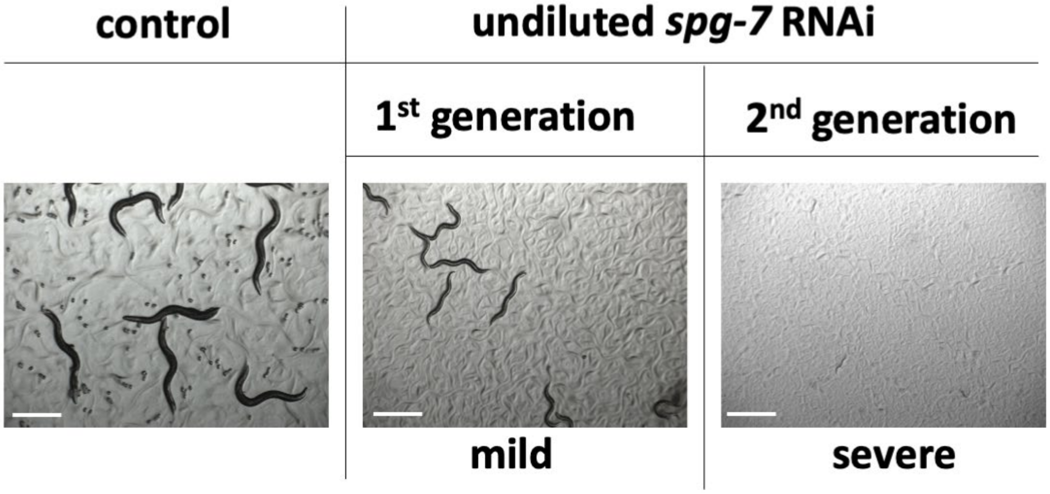

Figure S1. Different degrees of mitochondrial stress lead to different phenotypic readouts in *C. elegans*. Representative pictures of mild and strong phenotypes obtained in our screen of knockdown of mitochondrial proteins. HT115 bacteria expressing dsRNA targeting *spg-7* were fed to N2 (wild-type) animals in undiluted form, from the time of hatching for one (middle picture, mild) or two (right picture, severe) generations. Animals were photographed 4 days after hatching at the same magnification. Scale bar 500 µm. Control worms were fed pL4440 (empty vector) expressing bacteria. When *spg-7* and *tag-61* are diluted 1:10 in the parental generation, similar mild phenotypes are obtained.

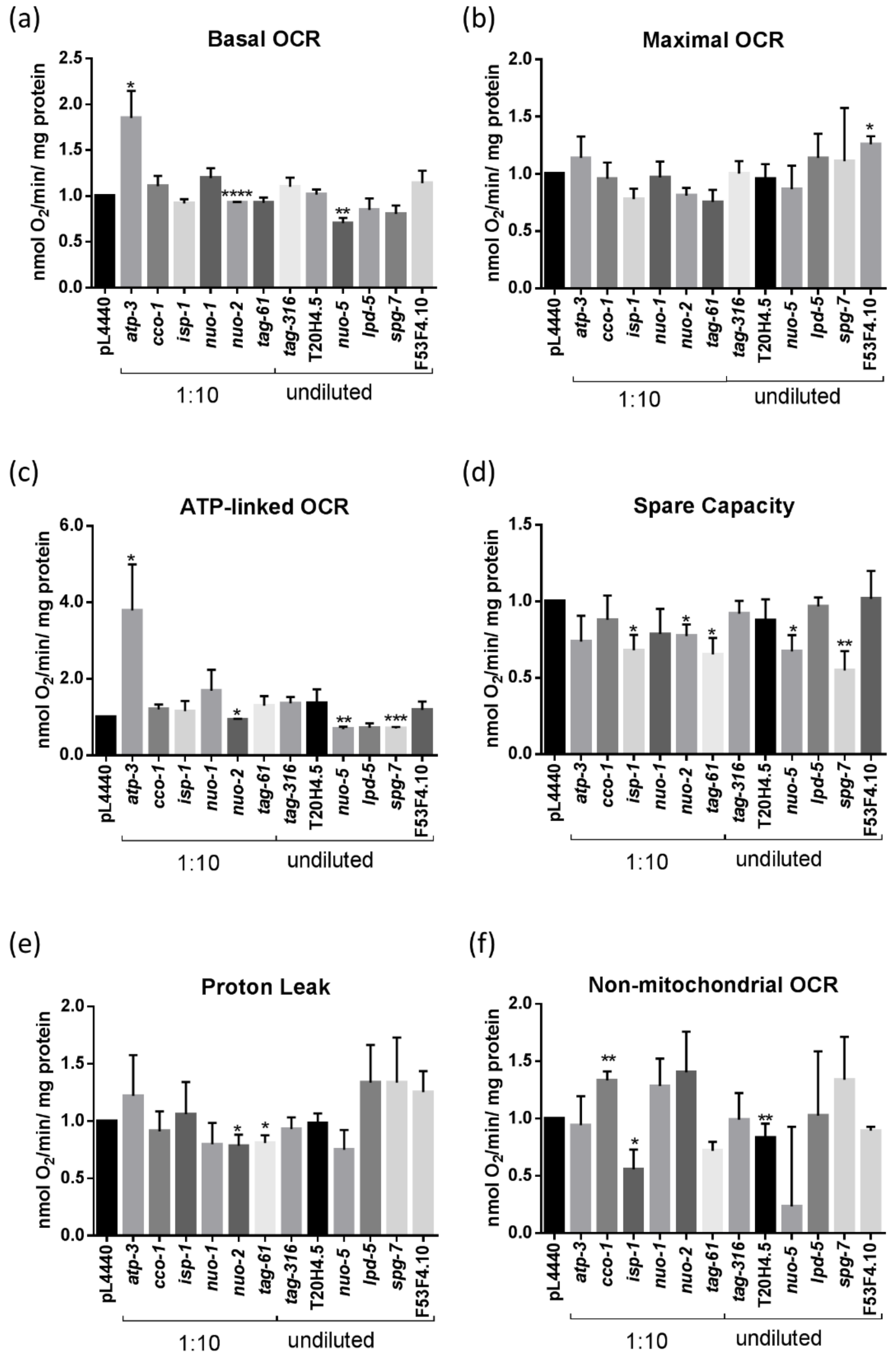

Figure S2. Seahorse measurements of different mitochondrial parameters. (**a**) Basal OCR, (**b**) Maximal OCR, (**c**) ATP-linked OCR, (**d**) Spare Capacity, (**e**) Proton Leak, and (**f**) Non-mitochondrial OCR. Refer to Supplementary Table SI and supporting information for complete information.

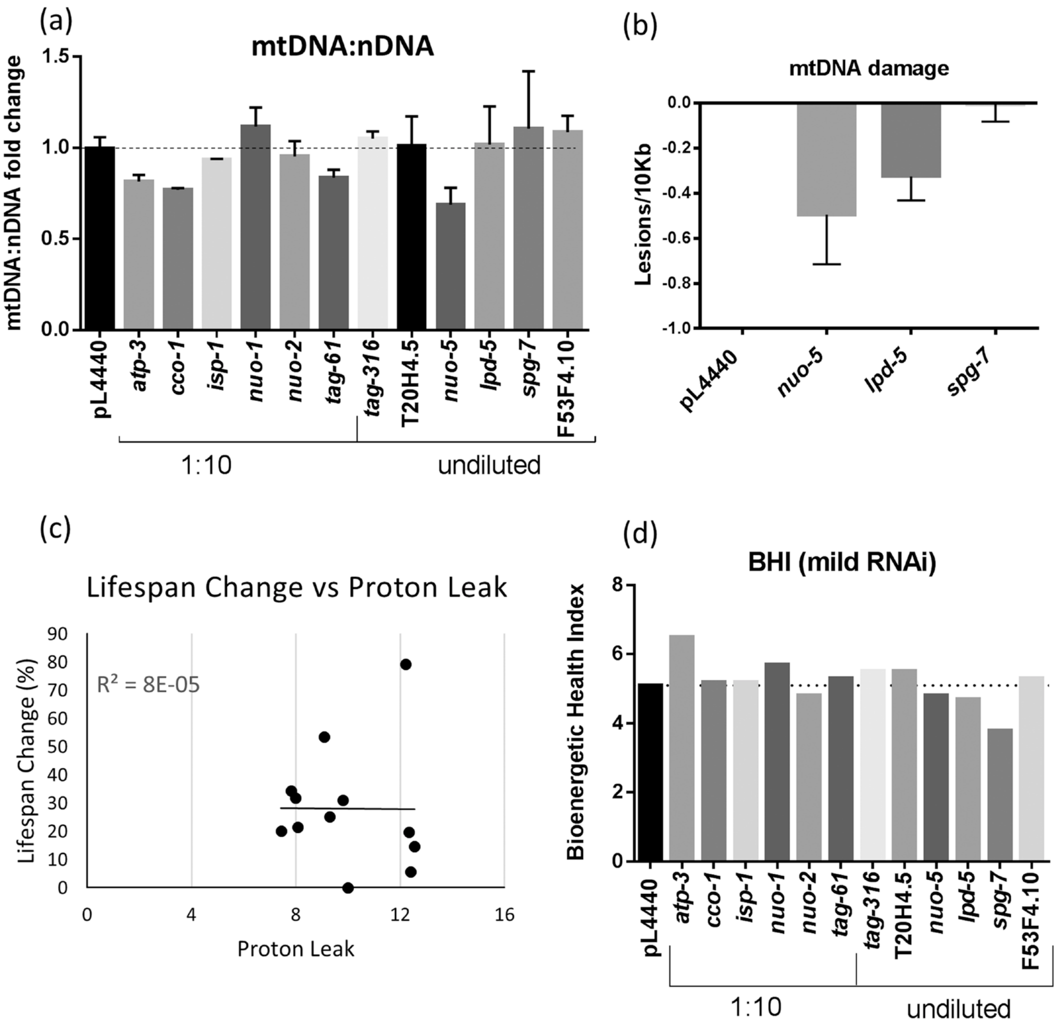

Figure S3. Additional mitochondrial analyses. Quantification of (**a**) mtDNA:nDNA ratio and (**b**) mtDNA damage normalized to control (empty vector pL4440) animals. The differences are not statistically significant. (**c**) % increase in mean lifespan plotted against proton leak in animals with mild suppression of different mitochondrial proteins. Each dot indicates an average value obtained from independent experiments with variable population sizes for each condition (for lifespan experiments an average of 60 animals per condition per replicate was used; for Seahorse experiment an average of 1500 animals per condition per replicate were employed; see Table S1 for details). (**d**) Bioenergetic Health Index (BHI) calculated for all clones under study (mild RNAi).

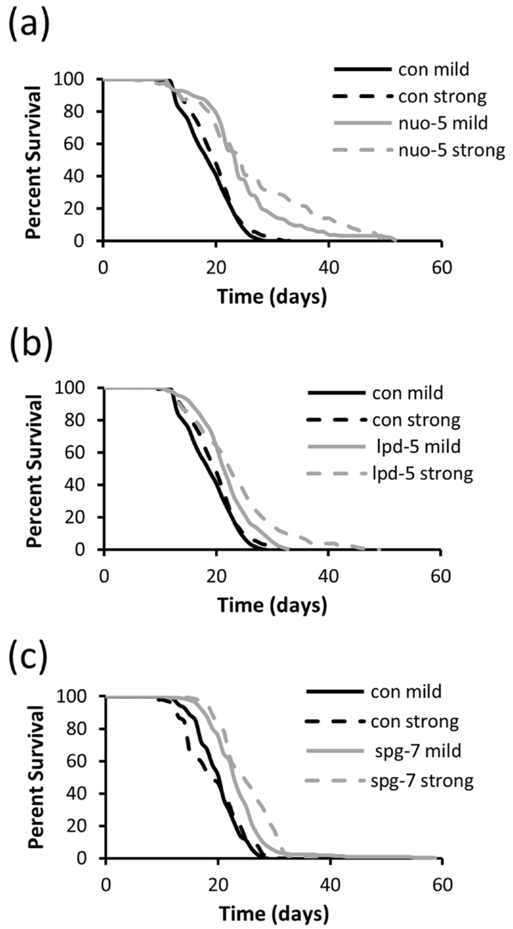

Figure S4. Lifespan is extended upon mild and strong suppression of three mitochondrial proteins. Kaplan-Meier survival curves of wild-type animals fed bacteria transformed with empty vector pL4440 (con) or with pL4440 vector expressing dsRNA targeting the indicated mitochondrial proteins. (**a**) *nuo-5,* (**b**) *lpd-5,* (**c**) *spg-7*. Mild suppression of the mitochondrial proteins is achieved in the parental (P0) generation of animals using undiluted RNAi, and strong suppression is observed in the second generation (F1). For all three clones the F1 animals arrest as L3 larvae and are therefore infertile. An average of 60 animals per condition was used in each replica and survival curves of pooled populations of animals coming from three independent replicas are shown.

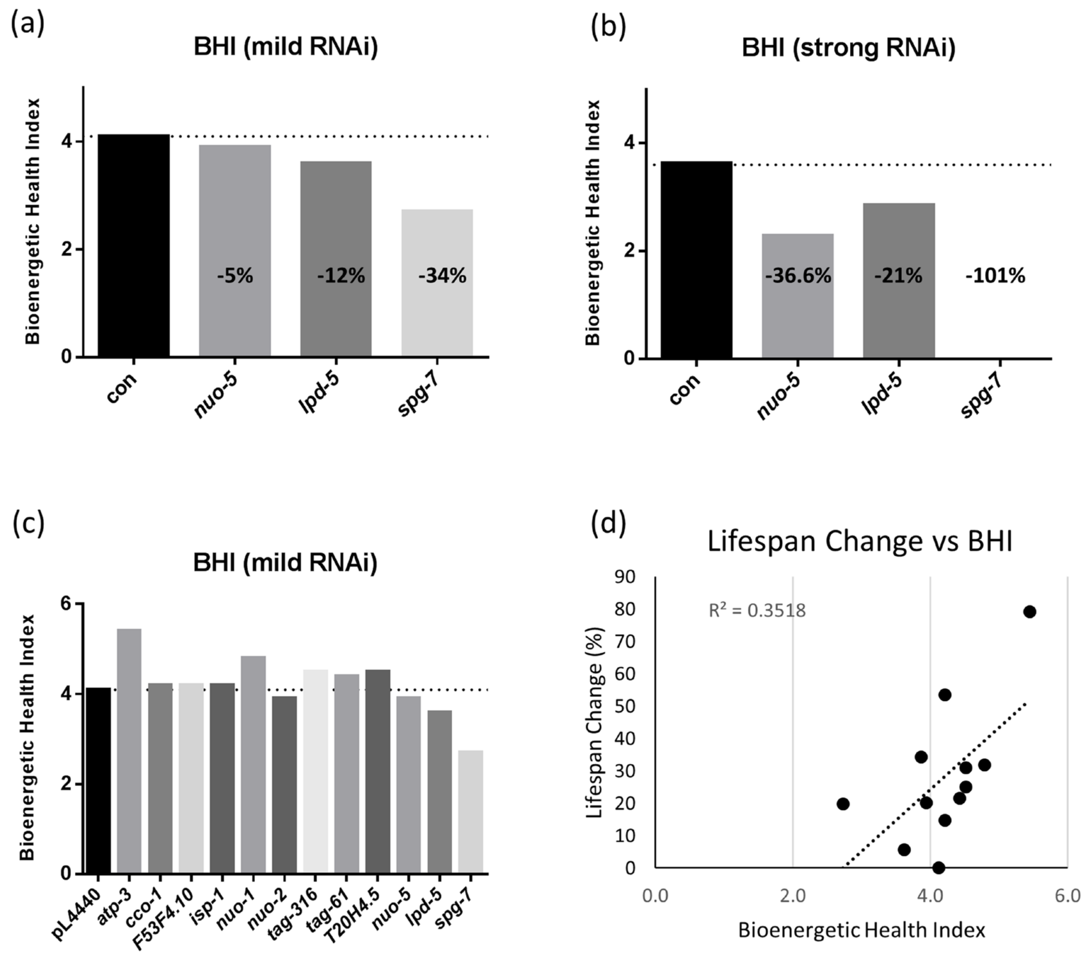

Figure S5. Alternative BHI calculation weighting proton leak more heavily. (**a**-**b**) Bioenergetic Health Index (BHI) using the alternative equation: BHI = log[(spare capacity)^2 x (ATP-linked OCR)^3]/[(non-mitochondrial OCR)^1 x (proton leak)^2], calculated for mild (**a**) and strong (**b**) RNAi-mediated suppression of *nuo-5, lpd-5* and *spg-7.* (**c**) % decrease of BHI compared to control is shown on the corresponding bar. BHI using the alternative equation calculated for all clones under study (mild RNAi). (**d**) Correlation plot between % increase in mean lifespan and BHIs (calculated with the alternative equation) in animals with mild suppression of different mitochondrial proteins.

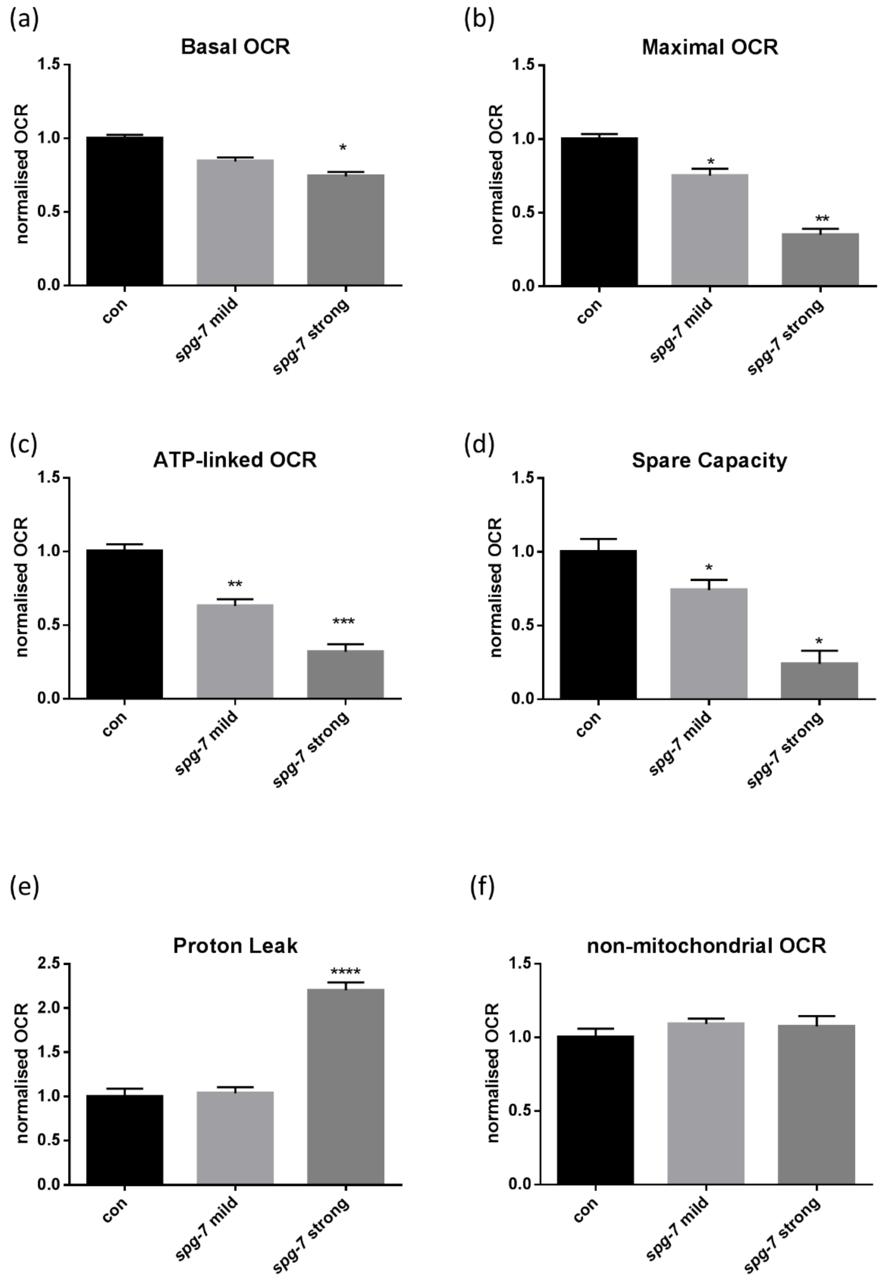

Figure S6. Mitochondrial respiratory parameters upon mild and strong suppression of *spg-7*. Comparison of respiratory parameters upon *spg-7* RNAi (mild and strong), normalized to the respective controls (stage-matched animals treated with empty vector pL4440). (**a**) Basal OCR, (**b**) maximal OCR, (**c**) ATP-linked OCR, (**d**) spare respiratory capacity, (**e**) proton leak and (**f**) non-mitochondrial OCR.
